# Morphological and Physiological Responses to Drought Stress of Three Species of the *Zygophyllum* Genus and a Comprehensive Evaluation of Their Drought Resistance

**DOI:** 10.3390/plants15152317

**Published:** 2026-07-28

**Authors:** Donghui Miao, Chengzhi Peng, Qi Ning, Yidan Yang, Zhibin Cui, Tongrui Song, Shuangfei Song, Pei Zhang, Peipei Jiao, Wenjuan Huang

**Affiliations:** 1 Xinjiang Production and Construction Corps Key Laboratory of Protection and Utilization of Biological Resources in Tarim Basin, College of Life Science, Tarim University, Alar 843300, China; mdh1216g@163.com (D.M.); orangeczpeng16@163.com (C.P.); ningqi20000423@163.com (Q.N.); yyd202510@163.com (Y.Y.); songtr20001209@163.com (T.S.); shuangfeisong@163.com (S.S.); 16609070757@163.com (P.Z.); 2 College of Horticulture and Forestry, Tarim University, Alar 843300, China; 17399975214@163.com

**Keywords:** *Zygophyllum*, drought stress, morphological, physiological characteristics, comprehensive evaluation

## Abstract

Drought stress triggers significant adaptive responses in plant morphology and physiology. As a representative xerophytic genus, *Zygophyllum* possesses distinct drought-adaptive traits in morphology, anatomical structure and physiological metabolism. This study examined the responses of three *Zygophyllum* species (*Zygophyllum fabago, Zygophyllum brachypterum,* and *Zygophyllum obliquum*) to progressive osmotic stress induced by 20% PEG-6000 over 28 days under controlled conditions. Among the three species, *Z. brachypterum* displayed the greatest tolerance, as evidenced by its lowest wilting rate, longest root elongation, and more stable leaf anatomical traits under stress, despite a general decrease in all measured structural parameters across species. Physiologically, *Z. brachypterum* showed the smallest reduction in leaf water content (5.99% vs. 13.51% in *Z. fabago* and 20.31% in *Z. obliquum*), the smallest increase in relative electrolyte leakage (126.72% vs. 172.84% and 162.91%, respectively), lower excised-leaf water loss, and sustained higher photosynthetic rates during later stress stages. It also accumulated the lowest levels of total reactive oxygen species (ROS) and malondialdehyde (MDA), alongside the highest proline content and the highest activities of superoxide dismutase (SOD), peroxidase (POD), and catalase (CAT). Based on a comprehensive evaluation integrating multiple morphological, anatomical, photosynthetic, antioxidant, and osmotic-adjustment indicators through principal component analysis and membership function analysis, the drought-resistance ranking at the seedling stage was determined as *Z. brachypterum* > *Z. fabago* > *Z. obliquum*. The superior tolerance of *Z. brachypterum* was associated with its more stable leaf structure, enhanced water retention, sustained late-stage photosynthesis, and stronger antioxidant and osmotic adjustment capacities. These findings provide physiological insights into PEG-induced osmotic stress tolerance in *Zygophyllum* seedlings.

## 1. Introduction

The continued intensification of global warming has led to significant shifts in the spatiotemporal distribution patterns of extreme drought events. As one of the most destructive natural disasters globally, drought has exhibited marked increases in frequency, duration, and affected area, particularly in arid and semi-arid regions and marginal monsoon-climate zones [1]. From a plant physiology perspective, drought stress inhibits plant growth and development by triggering associated physiological and biochemical responses [2,3]. A sharp decline in ambient humidity leads to excessive water loss within plants, resulting in decreased relative water content (RWC) and turgor pressure [4]. Drought stress severely restricts plant growth and reproduction and can be classified as mild, moderate, or severe based on RWC [5]. This physiological imbalance severely compromises vegetation health by inducing oxidative stress, causing membrane lipid peroxidation, and impairing metabolic functions, ultimately leading to tissue necrosis and plant mortality [6,7]. Beyond individual plant health, the frequent occurrence of extreme drought events destabilizes agro-ecosystems and poses a substantial threat to agricultural productivity and food security [8], making drought a core environmental constraint on plant growth and reproduction.

To cope with water deficit, plants have evolved sophisticated defense mechanisms. Upon sensing water deficit signals, plants initiate stomatal closure to minimize transpirational water loss; however, this process concurrently disrupts photosynthetic electron transport, thereby triggering excessive accumulation of reactive oxygen species (ROS) [9]. Meanwhile, water deficit induces osmotic potential imbalance, which in turn results in cellular dehydration [10], impairs plasma membrane integrity, and exacerbates the degree of membrane lipid peroxidation [11,12]. As drought stress persists, the photosynthetic efficiency of photosystem II (PSII) decreases significantly, and its photosynthetic electron transfer rate (ETR) is inhibited, which further damages the oxygen-evolving complex and reaction centers of PSII complexes [13]. To mitigate these drought-induced physiological injuries, plants have evolved a series of defense mechanisms. They initiate osmotic adjustment by accumulating compatible solutes to lower cellular osmotic potential, thereby enhancing water retention capacity and maintaining cell turgor [14]. During this process, plants also synthesize large quantities of phenolic compounds and other secondary metabolites [15], which not only efficiently scavenge excess ROS but also alleviate oxidative damage to the photosynthetic apparatus, ultimately improving plant drought resistance [16,17]. Collectively, this integrated physiological and biochemical response demonstrates that drought perception drives sequential changes in stomatal and photosynthetic traits, accompanied by ROS-mediated signaling pathways that synergistically coordinate antioxidant defense, osmotic adjustment, and metabolic regulation to enhance drought tolerance.

Beyond these physiological adjustments, drought-tolerant plants have evolved distinct adaptive characteristics to cope with arid environments, and these adaptive traits are mainly achieved through the optimization of organ morphology and cellular structure, which collectively enhance water acquisition capacity, minimize water loss, and maintain normal physiological functions [18,19]. Root architecture plasticity is a core adaptive strategy for drought foraging: increased root elongation expands access to deep soil moisture and elevates water uptake efficiency [20]. Leaves function as the principal organs for plant transpiration. Under drought stress, they display significant adaptive changes in morphology and structure, mainly including decreased specific leaf area [21], enhanced leaf succulence [22], elevated leaf reflectance [23], more distinct leaf lobation [24], and adaptive modifications in stomatal size and density [25]. Through the synergistic regulation of these morphological adaptations, drought-tolerant species achieve a higher water use efficiency (WUE), enabling them to maximize carbon gain with minimal water expenditure.

Desert xerophytes are ideal models for studying plant drought adaptation because they have evolved stable and efficient adaptive traits under long-term natural selection. *Zygophyllum* comprises approximately 150 species, making it the largest genus in the family Zygophyllaceae. It is widely distributed across the eastern Mediterranean coast, Central Asian countries and southern Africa and Australia [26]. *Zygophyllum fabago*, *Zygophyllum brachypterum*, and *Zygophyllum obliquum* are three typical desert xerophytes in the genus *Zygophyllum*. These closely related species are phylogenetically clustered and highly correlated in morphological adaptive traits and niche differentiation, exhibiting multiple stress resistances including drought tolerance, wind erosion resistance, and salt-alkali tolerance, thus playing vital roles in maintaining the structure and stability of arid desert ecosystems [27]. To date, research on *Zygophyllum* has mainly concentrated on taxonomy, phylogeny [26], stress physiological mechanisms [28,29,30], secondary metabolites [31] and medicinal value [32]. Among congeneric species, most studies have overwhelmingly focused on *Zygophyllum xanthoxylum*, while other related species remain poorly investigated. Furthermore, existing comparative studies are limited to discrete physiological and biochemical assays under drought, while systematic multi-dimensional comparisons of morphological, anatomical and physiological adaptive traits, as well as interspecific divergence in drought response strategies among congeneric relatives, remain scarce. We therefore hypothesize that xerophytic *Zygophyllum* species possess distinct drought tolerance strategies. To validate this hypothesis, we selected three sympatric *Zygophyllum* species (*Z. fabago*, *Z. brachypterum*, and *Z. obliquum*) and subjected their seedlings to progressive osmotic stress simulated by 20% PEG-6000 over a 28-day culture period under strictly controlled environments. A full spectrum of functional traits covering seedling morphology, leaf anatomical structure, water physiology, photosynthetic gas exchange, antioxidant metabolism and osmotic adjustment were dynamically quantified along the drought stress gradient. Principal component analysis combined with the fuzzy membership function evaluation was further adopted to quantify interspecific differences in comprehensive drought resistance and identify dominant drought-adaptive traits. This work fills the research gap regarding multi-dimensional interspecific differentiation of drought adaptation within *Zygophyllum*. Our integrated morphological-anatomical-physiological comparative framework distinguishes this study from previous single-indicator physiological tests, and the quantitative comprehensive evaluation model robustly verifies the interspecific divergence of drought adaptive strategies hypothesized herein. Collectively, these results reveal core physiological mechanisms underlying PEG-simulated osmotic stress tolerance in *Zygophyllum* seedlings, deliver credible theoretical support for drought-resistant forage germplasm screening in arid zones, and offer practical references for optimizing vegetation allocation during desertification restoration.

## 2. Results

### 2.1. Morphological Characteristics of Three Zygophyllum Species Under Different Drought Stress Times

The morphological indicators of the three *Zygophyllum* species were measured before and after drought stress. The results showed that before drought stress, the three *Zygophyllum* species exhibited significant differences in plant height (PH) and root length (RL), with *Z. fabago* exhibiting the greatest PH (8.56 cm), *Z. brachypterum* possessing the longest RL (5.63 cm), and *Z. obliquum* displaying lower values of both indicators (Supplementary Table S1).

Under drought stress, all three *Zygophyllum* species exhibited varying degrees of wilting with prolonged stress duration (Figure 1A). Their wilting rate (WR) significantly increased and reached maximum values at 28 days; at which point, *Z. obliquum* and *Z. brachypterum* showed the highest (84.32%) and lowest (64.99%) WRs, respectively (Figure 1B). The PH of all three *Zygophyllum* species showed significant decreases with prolonged stress and reached the lowest values at 28 days, with *Z. fabago*, *Z. brachypterum*, and *Z. obliquum* showing decreases of 54.81%, 46.13%, and 70.32%, respectively, compared with their controls (Figure 1C). Under drought stress, the RL of all three species increased to varying degrees, reaching the maximum values at 28 days. Consistent with this, *Z. brachypterum* exhibited the most pronounced root elongation (8.52% increase vs. control; Figure 1D), suggesting a superior capacity for resource acquisition under drought.

Before drought stress treatment, the three *Zygophyllum* species exhibited differences in stomatal density (SD) in the following order: *Z. fabago* (283 units/mm^2^) > *Z. brachypterum* (204 units/mm^2^) > *Z. obliquum* (142 units/mm^2^). No significant differences in stomatal opening rate (SOR) were found among the three species before stress treatment (Figure 2A).

Under prolonged drought stress, *Z. fabago* and *Z. obliquum* showed an initial increase followed by a subsequent decrease in SD. At 28 days of stress, the SD of *Z. fabago* dropped below the CK level, and the remaining two species reached their maximum SD at 21 days of stress. By contrast, *Z. brachypterum* exhibited an SD trend of initially increasing, then decreasing, and finally growing under drought stress, achieving its highest SD (404 units/mm^2^) at 28 days. The maximum SDs of *Z. fabago*, *Z. brachypterum*, and *Z. obliquum* were significantly higher by 34.31% (21 days), 98.00% (28 days), and 38.70% (21 days), respectively, compared with their controls (Figure 2B).

Under drought stress, the SOR of all three species gradually declined and reached the lowest value at 28 days, with *Z. fabago*, *Z. brachypterum*, and *Z. obliquum* showing decreases of 52.61%, 41.44%, and 52.95%, respectively, compared with their controls. At this stage, *Z. brachypterum* maintained a relatively high SOR, and *Z. obliquum* had the lowest SOR (Figure 2C).

### 2.2. Leaf Anatomical Structure of Three Zygophyllum Species Under Different Drought Stress Times

With prolonged drought stress, the three *Zygophyllum* species exhibited alterations in upper epidermal cell thickness (UEC), lower epidermal cell thickness (LEC), upper cuticle thickness (UC), lower cuticle thickness (LC), palisade tissue thickness (PT), spongy tissue thickness (ST), leaf thickness (LT), and midrib vascular bundle area (VB) (Supplementary Table S2).

Observation of the leaf anatomical structures of the three *Zygophyllum* species under drought stress revealed varying degrees of structural modifications, collectively manifesting as gradual thinning and curling. Overall, *Z. brachypterum* exhibited the smallest reductions in palisade tissue, spongy tissue, leaf thickness and vascular bundle area, while *Z. obliquum* showed the largest declines in palisade tissue thickness and leaf thickness (Figure 3).

Before drought stress treatment, the three *Zygophyllum* species exhibited differences in UEC, LEC, UC, and LC. *Z. brachypterum* displayed the largest UEC (32.89 μm), LEC (33.05 μm), and UC (7.07 μm), and *Z. obliquum* had the thickest LC (6.63 μm).

Under drought stress, the UEC, LEC, UC, and LC of the three *Zygophyllum* species generally showed a decreasing trend (Figure 4A–D). When the UEC reached its lowest value, *Z. fabago*, *Z. brachypterum*, and *Z. obliquum* decreased by 4.12% (28 d), 13.43% (28 d), and 8.15% (21 d), respectively, compared to their controls (Figure 4A); the LEC of all three species dropped to the lowest levels at 28 days of drought stress, with *Z. fabago*, *Z. brachypterum*, and *Z. obliquum* decreasing by 3.37%, 13.24%, and 6.93%, respectively, compared to their controls (Figure 4B); the UC of *Z. fabago* and *Z. obliquum* returned to control levels at 28 days of drought stress, showing no significant differences, while *Z. brachypterum* was significantly lower than its control, with a 42.32% reduction compared to the control (Figure 4C); the LC of *Z. fabago* returned to the control level at 28 days of drought stress, with no significant difference, whereas *Z. brachypterum* and *Z. obliquum* were significantly lower than their controls, decreasing by 35.24% and 43.47%, respectively, compared to their controls (Figure 4D).

Before drought stress, the three *Zygophyllum* species exhibited differences in PT, ST, LT, and VB: *Z. obliquum* had the most significant PT (187.26 μm) and LT (369.34 μm), but the smallest VB (15,075.97 μm^2^). At the same time, *Z. fabago* displayed the greatest ST (188.39 μm).

Under drought stress, the PT, ST, LT, and VB of leaves in the three *Zygophyllum* species generally decreased. When PT reached its lowest values, *Z. fabago*, *Z. brachypterum*, and *Z. obliquum* decreased by 61.05%, 49.31%, and 82.45%, respectively, compared to their controls (Figure 4E); when ST reached its lowest values, *Z. fabago*, *Z. brachypterum*, and *Z. obliquum* decreased by 64.12%, 57.11%, and 70.50%, respectively, compared to their controls (Figure 4F); when LT reached its lowest values, *Z. fabago*, *Z. brachypterum*, and *Z. obliquum* decreased by 59.41%, 51.72%, and 71.74%, respectively, compared to their controls (Figure 4G); when VB reached its lowest values, *Z. fabago*, *Z. brachypterum*, and *Z. obliquum* decreased by 62.82% (28 d), 41.41% (28 d), and 47.60% (21 d), respectively, compared to their controls (Figure 4H).

Overall, all leaf anatomical indices declined to varying degrees in the three species under progressive drought stress. Among them, *Z. brachypterum* showed the smallest decline in PT, ST, LT and VB, while *Z. obliquum* exhibited the largest reduction in PT and LT.

### 2.3. Water Physiological Characteristics of Three Zygophyllum Species Under Different Drought Stress Times

With prolonged drought stress, the three *Zygophyllum* species exhibited alterations in water content (WC) and relative electrical conductivity (REC) (Supplementary Table S3) and differences in the rate of excised-leaf water loss (RWL) of detached leaves (Supplementary Tables S3 and S4).

Prior to drought stress treatment, WC and REC differed among the three species, with the highest WC (96.39%) detected in *Z. obliquum* and the highest REC (2.99%) in *Z. fabago.* Under drought stress, the WC of all three *Zygophyllum* species reached its lowest values at 28 days, with *Z. fabago*, *Z. brachypterum*, and *Z. obliquum* showing decreases of 13.51%, 5.99%, and 20.31%, respectively, compared with their controls (Figure 5A). The REC of all three species increased continuously and peaked at 28 days, with *Z. fabago*, *Z. brachypterum*, and *Z. obliquum* showing increases of 172.84%, 126.72%, and 162.91%, respectively, compared with their controls (Figure 5B). Drought stress significantly increased the REC in all three *Zygophyllum* species compared to the well-watered control (*p* < 0.05). Notably, *Z. brachypterum* exhibited the lowest relative electrolyte conductivity at all stress time points. For excised-leaf RWL, all three species showed significantly higher water loss at each successive measurement point under drought. After 8 h of dehydration, *Z. obliquum* exhibited the highest RWL (75.84%), which was substantially higher than those of *Z. fabago* (47.35%) and *Z. brachypterum* (41.16%) (Figure 5C), indicating poorer leaf water retention capacity in *Z. obliquum*.

### 2.4. Photosynthetic Physiological Characteristics of Three Zygophyllum Species Under Different Drought Stress Times

Photosynthetic parameters were measured in the three *Zygophyllum* species under well-watered and drought conditions. Under well-watered conditions, significant differences in net photosynthetic rate (Pn), transpiration rate (Tr), stomatal conductance (Gs) and WUE were detected among the three species (Supplementary Table S5). *Z. fabago* exhibited the maximum values of Pn (3.54 μmol CO_2_/(m^2^·s)), Tr (2.73 mmol H_2_O/(m^2^·s)) and Gs (0.19 mol H_2_O/(m^2^·s)), while *Z. obliquum* had the highest WUE (1.91 μmol CO_2_/mmol H_2_O).

With the prolongation of drought stress, Pn in all three species continuously decreased and reached the minimum at 28 d, with declines of 73.42%, 73.54%, and 73.71% in *Z. fabago*, *Z. brachypterum*, and *Z. obliquum*, respectively, relative to the control (Figure 6A).

Consistent with this trend, Tr and Gs of the three species both peaked at 14 d under drought treatment. Compared with the control, Tr increased by 28.91%, 74.24%, and 84.01%, and Gs increased by 55.91%, 83.32%, and 139.14% in the three species in sequence. Subsequently, Tr and Gs declined sharply to the lowest levels at 28 d. At this time, Tr decreased by 86.84%, 69.13%, and 90.61%, and Gs decreased by 91.54%, 85.81%, and 77.22% in *Z. fabago*, *Z. brachypterum*, and *Z. obliquum*, respectively (Figure 6B,C).

Under drought stress, divergent WUE patterns were observed among the three species. In *Z. fabago* and *Z. obliquum*, WUE increased significantly during the late stress period, with rises of 104.61% and 25.12% above their respective controls (Figure 6D). This elevation in WUE is primarily attributed to a more pronounced reduction in Tr relative to the decline in net Pn, a typical consequence of progressive stomatal closure aimed at conserving water under severe drought. In contrast, *Z. brachypterum* exhibited an overall decrease in WUE, reaching its lowest point at 21 days. This pattern suggests that *Z. brachypterum* maintained a higher relative stomatal conductance or photosynthetic activity for a longer duration, prioritizing carbon assimilation over strict water conservation until later stress stages, which may constitute a different drought-coping strategy.

### 2.5. Antioxidant Physiological Characteristics of Three Zygophyllum Species Under Different Drought Stress Times

Under well-watered conditions, the three *Zygophyllum* species exhibited similar basal levels of antioxidant physiological parameters (Supplementary Table S6). After drought stress treatment, oxidative damage indicators and osmotic regulatory substances showed a continuous increasing trend, while the activities of the three antioxidant enzymes consistently displayed a pattern of initial increase followed by decrease, peaking at 14 days of stress and being significantly higher than those in control plants.

The total intracellular ROS content of all three species reached the maximum values at 28 days, with *Z. brachypterum* showing the lowest (616,450.70 fluorescence intensity/mg protein) and *Z. obliquum* showing the highest (735,563.30 fluorescence intensity/mg protein) (Figure 7A). The MDA content of all three species peaked at 28 days, with *Z. fabago*, *Z. brachypterum*, and *Z. obliquum* increasing by 202.81%, 136.43%, and 242.24%, respectively, compared with their controls (Figure 7B). The Pro content of all three species reached the maximum values at 28 days, with *Z. obliquum* showing the lowest (680.30 μg/g) and *Z. brachypterum* showing the highest (987.70 μg/g) (Figure 7C).

The POD activity of all three species peaked at 14 days, with *Z. obliquum* exhibiting the lowest (4996.60 U/g) and *Z. brachypterum* exhibiting the highest (8848.20 U/g). The POD activity declined gradually across all three species. At 28 days, *Z. brachypterum* (1235.70 U/g) showed a significantly higher POD activity than *Z. fabago* (847.50 U/g) and *Z. obliquum* (590.70 U/g). At this stage, the POD activities in *Z. brachypterum* and *Z. fabago* remained significantly higher than those in their controls. Although *Z. obliquum*’s POD activity was higher than that of its control, the difference was not significant (Figure 7D).

The SOD activity of all three species peaked at 14 days, with *Z. obliquum* showing the lowest (169.80 U/g) and *Z. brachypterum* showing the highest (180.60 U/g). Afterwards, the SOD activity gradually declined, and at 28 days of drought stress, the order was *Z. brachypterum* (97.80 U/g) > *Z. fabago* (87.70 U/g) > *Z. obliquum* (80.30 U/g). Nevertheless, SOD activity in all three species remained higher than their respective controls (Figure 7E).

The CAT activity of all three species peaked at 14 days, with *Z. obliquum* exhibiting the lowest (315.60 U/g) and *Z. brachypterum* exhibiting the highest (366.20 U/g). Afterwards, the CAT activity progressively decreased and became significantly lower than that of the controls at 28 days in the order of *Z. brachypterum* (114.80 U/g) > *Z. fabago* (101.70 U/g) > *Z. obliquum* (85.80 U/g) (Figure 7F).

### 2.6. Principal Component Analysis (PCA) of 26 Indicators of Three Zygophyllum Species Under Drought Stress

PCA was performed on 26 drought stress-related indicators of three *Zygophyllum* species, and the first five principal components with eigenvalues greater than 1 collectively explained 91.109% of the total variation, adequately reflecting the main information of all measured indices. PC1 accounted for 53.414% of the variance, dominated by most morphological growth traits, water physiological parameters, photosynthetic indices, and oxidative stress indicators, comprehensively mirroring the overall growth performance, water retention capacity, photosynthetic capacity and systemic physiological response level of plants under drought stress; PC2 explained 18.227% of the variance, closely associated with antioxidant enzyme activities, proline content and stomatal gas exchange parameters, representing the plant’s antioxidant defense capacity, osmotic adjustment ability and the coordination between protective physiological systems and stomatal behavior under drought; PC3 contributed 9.333% of the variance, strongly correlated with root length, stomatal density, upper cuticle thickness, water use efficiency and proline content, integrating multiple functional traits involved in root water absorption, leaf structural water retention, water use strategy and osmotic adjustment; PC4 and PC5 had comparatively weaker explanatory power, accounting for 6.135% and 4.000% of the total variance, respectively. They were mainly associated with fine-scale variation in leaf anatomical structure (e.g., palisade tissue thickness) and partial morphological traits, providing Supplementary Information for interpreting interspecific differences in leaf structural adaptation beyond the first three principal components (Table 1).

### 2.7. Comprehensive Evaluation of the Drought Resistance of Three Zygophyllum Species

For the comprehensive drought resistance evaluation, the 26 drought stress-related indicators were first reduced to five principal components via PCA, and the principal component score of each species on each component was calculated. Based on the scores of the five extracted principal components, the membership function values U(PC1) to U(PC5) and the comprehensive drought resistance evaluation index of the three *Zygophyllum* species were calculated. The *D* value, which quantifies the overall drought resistance performance, followed the descending order of *Z. brachypterum* (0.649) > *Z. fabago* (0.592) > *Z. obliquum* (0.535), indicating that *Z. brachypterum* exhibited the strongest comprehensive drought resistance among the three tested species (Table 2).

## 3. Discussion

Drought stress, a principal abiotic constraint on plant growth and development, drives the evolution of specialized morphological adaptations essential for the persistence and reproductive success of plant populations in arid environments [33]. These adaptations, primarily manifested as growth modulation and structural remodeling of root and leaf tissues, serve as direct phenotypic indicators of a plant’s capacity to cope with water deficits [34]. In this study, all three species exhibited growth inhibition under drought conditions, whereas *Z. brachypterum* displayed significantly stronger drought tolerance, as evidenced by slighter leaf wilting, more stable plant height, and more pronounced root elongation. The pronounced root elongation in *Z. brachypterum* aligns with the classic xerophytic strategy of expanding soil exploration volume to compensate for surface water deficit, supporting sustained water uptake under progressive stress [35]. Compared with the other two species, *Z. brachypterum* maintained better structural integrity of aboveground tissues while sustaining root proliferation, forming a highly coordinated growth pattern between shoots and roots under water deficit [36]. This coordinated morphological adjustment helps mitigate the inherent trade-off between maintaining photosynthetic organ function and improving underground water foraging capacity [37]. Such favorable morphological traits allow *Z. brachypterum* to maintain basic growth and physiological activities under drought, which underpins its better drought performance relative to congeneric species.

As the primary organs for material and energy exchange between plants and the external environment, leaves exhibit exceptional sensitivity to environmental changes. Under drought stress, the UEC, LEC, UC and LC of all three species showed overall declining trends, and a similar decreasing trend was also observed in *Cynanchum thesioides* under drought stress [38]. These leaf structural parameters are closely associated with plant drought adaptation, and our results indicate that drought stress generally restrains the development of these anatomical traits. With the intensification of drought stress, LT, ST, PT, and VB gradually decreased in all three species, which may be attributed to continuous leaf water loss and inhibited leaf growth and vascular development [39]. *Z. brachypterum* showed temporary increases in ST, PT, and VB during the early to middle stages of stress. According to the well-documented structure-function relationship of leaf anatomy, thickened mesophyll tissues may support higher water storage capacity, and expanded vasculature may contribute to more efficient resource transport; these structural adjustments may thus partially buffer the negative impacts of drought [40]. At the late stage of drought stress, all four anatomical parameters declined across the three species, yet *Z. brachypterum* retained significantly higher values than the other two species. This pattern indicates that *Z. brachypterum* exhibits greater anatomical plasticity under water deficit, which may underlie its superior drought adaptive capacity relative to its congeners. It should be noted, however, that the actual changes in water storage and transport function were not directly quantified in this study, and the functional benefits of these anatomical changes remain to be verified.

Leaf relative water content (WC) and rate of excised-leaf water loss (RWL) are key indicators reflecting plant water status and dehydration resistance. Marshall et al. [41] reported that drought-tolerant species usually maintain higher WC and lower RWL under stress to sustain normal physiological functions. Liu et al. [42] suggested that plants with more stable water retention capacity exhibit stronger drought adaptability. Under water stress, changes in these two parameters directly reflect the degree of water deficit and tissue tolerance to dehydration [43,44]. Previous studies have demonstrated that the WC in typical desert shrubs, including *Z. xanthoxylum* [45], *Hedysarum scoparium* [46], *Ammopiptanthus mongolicus* [47], and *Caragana microphylla* [48], decreases significantly with prolonged drought stress. In the present study, with increasing drought stress, leaf WC decreased while RWL increased progressively in all three *Zygophyllum* species; *Z. brachypterum* consistently maintained the lowest RWL under each drought intensity, demonstrating that this species possesses stronger leaf water-holding capacity and higher dehydration resistance, and can thus sustain a more favorable leaf water status under water deficit compared with the other two congeneric species.

Drought stress typically suppresses photosynthetic activity (Pn) and organic matter accumulation [49,50]. In this study, the Pn of all three *Zygophyllum* species showed an initial increase followed by a continuous decline with prolonged 20% PEG-6000 stress, reaching minimum values at 28 days with similar relative reductions (73.42–73.71%). The driver of this transient early Pn increase remains unclear without targeted measurements of photosynthetic limitation. This transient elevation under mild drought likely represents a short-term adaptive response, in which plants optimize stomatal behavior to balance CO_2_ uptake and water loss, rather than a genuine enhancement of photosynthetic capacity [51]. As drought intensified, leaf gas exchange was progressively impaired, leading to the sustained decrease in Pn [52]. Since the relative contributions of stomatal and non-stomatal limitations were not distinguished in this study, the underlying mechanisms driving early photosynthetic fluctuation and late-stage decline remain to be verified using intercellular CO_2_ concentration and chlorophyll fluorescence analyses. Interspecific divergence in WUE likely reflects fundamentally different drought adaptation strategies among the three species. *Z. fabago* and *Z. obliquum* showed markedly elevated late-stage WUE (104.61% and 25.12% increases, respectively), driven by a larger reduction in transpiration than in photosynthesis. This pattern is consistent with an isohydric water-saving strategy where rapid stomatal closure reduces water loss at the cost of carbon gain, which can lead to carbon starvation under extended drought. Notably, *Z. brachypterum* exhibited a stronger initial increase in gas exchange performance and retained higher residual Pn at 28 days than its congeners, indicating superior photosynthetic stability and greater resistance to drought-induced photoinhibition. With respect to WUE, *Z. brachypterum* displayed an overall decreasing trend with moderate fluctuations. This pattern indicates that *Z. brachypterum* may prioritize sustained photosynthetic carbon gain over strict water conservation in its drought response [53]. This pattern aligns with an anisohydric dehydration tolerance strategy, where stomata remain relatively open to sustain photosynthesis rather than closing fully to raise WUE, as supported by its lower wilting rate, reduced membrane damage, higher antioxidant enzyme activity and higher proline content across stress stages [54]. All these traits represent core functional adaptations supporting cellular dehydration tolerance, with proline acting as both osmoprotectant and reactive oxygen species scavenger to stabilize cell structure under low water potential. This adaptive pattern matches observations of dominant desert shrubs in Central Asia [55] and the congeneric *Z. xanthoxylum*, which maintains photosynthesis under drought via osmotic adjustment rather than relying on high WUE [56]. As dynamic leaf water potential was not measured in this study, species cannot be definitively categorized into strict isohydric/anisohydric groups, and this strategy differentiation requires further verification.

Drought-induced overaccumulation of ROS is widely recognized as a core driver of cellular oxidative damage, with REC and MDA serving as standard indirect indicators of plasma membrane integrity and stress injury severity [57,58]. In this study, both REC and MDA increased progressively with stress intensification across all three *Zygophyllum* species. The gradual rise in REC appears consistent with observations in *Acacia mangium* under drought conditions [59], while the stress-dependent MDA accumulation aligns with previous findings in the congener *Z. xanthoxylum* [45], suggesting shared susceptibility to oxidative damage within the genus. MDA response patterns vary markedly across desert xerophytes: species such as *Atriplex canescens* show steady MDA elevation as stress intensifies [60], whereas highly tolerant xerophytes like *Tamarix chinensis* exhibit no persistent MDA accumulation, which is generally interpreted as reflecting strong constitutive membrane stability and antioxidant capacity [61]. Among the three studied species, *Z. brachypterum* showed the smallest increments in both REC and MDA, implying milder membrane damage and oxidative stress under drought. This advantage may be primarily driven by its more efficient antioxidant defense system. SOD, POD and CAT function synergistically to scavenge excess ROS: SOD converts superoxide radicals into less reactive H_2_O_2_, which is further decomposed into water and oxygen by POD and CAT [62]. Across all species, activities of these three enzymes exhibited an initial increase followed by a decline, which may correspond to transient adaptive upregulation followed by impaired cellular metabolism under severe drought. Notably, *Z. brachypterum* maintained significantly higher POD activity than its congeners across all stress stages, potentially contributing to more sustained ROS clearance. Nevertheless, as overall cellular metabolic activity was not directly quantified, the exact mechanisms underlying the late-stage reduction in antioxidant enzyme activity remain to be verified. In addition to enzymatic antioxidant defense, proline accumulation may also contribute to the superior membrane stability observed in *Z. brachypterum*. As a critical osmoprotectant closely associated with drought adaptation [63], proline is thought to serve dual protective roles: it lowers cellular osmotic potential to enhance water retention, and may also stabilize membrane structure to mitigate peroxidative damage, collectively reducing dehydration-induced cellular injury.

Alongside morphological, anatomical and photosynthetic adaptive traits, the robust antioxidant defense system and enhanced osmotic adjustment were closely associated with the stronger drought tolerance observed in *Z. brachypterum*. The multi-trait comprehensive evaluation further confirms the interspecific differentiation in drought resistance among the three *Zygophyllum* species. It should be noted that all associations reported here are based on observed phenotypic correlations. Future manipulative experiments are needed to verify the causal contributions of individual traits to drought tolerance.

## 4. Materials and Methods

### 4.1. Sampling Site Overview and Seed Collection Strategy

Plant materials were sampled from locations around the Tarim Basin (Xinjiang Uygur Autonomous Region, China) with the following geographical coordinates: 83°3′30.58″ E, 41°53′32.27″ N, altitude 1295 m; 79°49′14.69″ E, 41°6′49.81″ N, altitude 1209 m; 74°33′25.58″ E, 39°49′51.34″ N, altitude 2746 m (Supplementary Table S7, Figure 8). These three sampling sites, all located outside urban areas, naturally supported abundant populations of *Z. fabago*, *Z. brachypterum*, and *Z. obliquum*. All sampling sites are located in temperate desert climate zones characterized by abundant year-round sunshine, sparse rainfall and large diurnal temperature differences, with an annual mean precipitation of 50–120 mm, an annual mean temperature of 10–12 °C, and predominantly infertile, well-drained sandy or sandy loess soil (https://data.cma.cn/).

Seeds of each species were randomly collected from 40 individual plants across three sampling plots within a single representative natural population, with collection performed from July to September during the natural fruit maturation period. For each species, all seeds were sourced from the same natural population, and three dispersed sampling plots were established to ensure uniform sampling across the full distribution of the population and avoid sampling bias from concentrated collection in a single habitat. This sampling intensity was determined with reference to standard practices in wild plant ecophysiological research, and was designed to capture natural intraspecific variation, ensure the biological representativeness of samples, and support sufficient biological replicates for subsequent statistical analyses. Specifically, only uniformly mature, healthy seeds of consistent size were selected to minimize differences in initial seed vigor and quality, thereby reducing potential interference from maternal effects to the greatest extent possible; however, residual maternal environmental effects cannot be fully eliminated. After natural air-drying, the seeds were sieved and stored at 4 °C in a refrigerator for subsequent use. All seed collection procedures were conducted in strict accordance with local and national regulations governing the use of wild plant resources in China.

### 4.2. Drought Treatment of Zygophyllum Seedlings

Plump seeds of three species belonging to the genus *Zygophyllum* were selected, subjected to seed soaking pretreatment, and then sown on 10 g/L agarose medium for germination culture. After full expansion of seedling cotyledons, uniformly grown plants were selected and transplanted into pots. Each pot was filled with an equal dry weight of thoroughly mixed vermiculite-perlite substrate (3∶1, *v*/*v*) to ensure consistent total water-holding capacity across all replicates, with 9 seedlings per pot. Following transplantation, each pot was thoroughly irrigated until water overflowed from the bottom to ensure consistent initial water content of the substrate across all replicates. The seedlings were cultured under controlled conditions of a constant temperature of 25 °C, a 16 h photoperiod, and a photosynthetic photon flux density (PPFD) of 300 μmol·m^−2^·s^−1^. During the regular culture stage before stress treatment, each pot was quantitatively irrigated with 500 mL of deionized water at 10:00 a.m. every day.

When approximately 80% of seedlings reached the 10-leaf stage, simulated osmotic drought stress was initiated using PEG-6000. A 20% (*w*/*v*) PEG-6000 solution was prepared by dissolving accurately weighed PEG-6000 powder in deionized water at room temperature with continuous magnetic stirring until fully dissolved. The solution was left to stand for 24 h to stabilize osmotic potential before use. The initial osmotic potential of the 20% PEG-6000 solution was approximately −0.65 MPa, as validated in previous drought studies on congeneric *Zygophyllum* species [27]. To maintain stable osmotic stress, 20% PEG-6000 solution was replenished every 3 days to compensate for water loss via transpiration and evaporation.

The experiment was arranged in a completely randomized design (CRD), with five treatment groups consisting of 0 d (CK), 7 d, 14 d, 21 d and 28 d of drought stress for each species. Three independent biological replicates were set up for the entire experiment, with each replicate representing a complete, independent run of sowing, treatment and measurement conducted under identical controlled conditions. For each biological replicate, 6 pots (9 plants per pot) per sampling time point per species were initially prepared as backup units, to buffer against seedling mortality, pest damage and growth heterogeneity during the 28-day long drought treatment. Before formal sampling, pots meeting any of the following criteria were excluded as abnormal: (1) >10% seedling mortality; (2) average plant height deviating >20% from the group mean; (3) visible pest or disease symptoms. Three pots with uniform seedling growth were then randomly selected from the remaining qualified pots for all index measurements. Seedlings within the same pot were treated as subsamples rather than independent units, as they shared identical substrate conditions and stress intensity.

For statistical analysis of all traits, values from technical replicates were first averaged per leaf/seedling; values from subsampled seedlings within the same pot were then averaged to generate a pot-level value; the mean of the three selected pots within one biological replicate was calculated as the trait value for that replicate. Final results are expressed as mean ± standard deviation of the three independent biological replicates.

### 4.3. Determination Method of the Morphological Characteristics Index

At each sampling time point, sampling was performed strictly following the nested design described in Section 4.2: for each independent biological replicate, 3 seedlings with uniform growth were randomly selected from the 3 pre-selected qualified pots, resulting in 9 measured seedlings per biological replicate. It should be noted that the use of seedlings with relatively uniform initial growth status was solely to minimize pre-existing developmental differences prior to stress treatment, rather than to artificially eliminate natural biological variation. PH was measured using a standard ruler, and RL was determined using a digital vernier caliper to the nearest 0.01 mm.

At each sampling time point, whole-seedling phenotypes were photographed to document wilting symptoms, and the number of wilted seedlings in each pot was counted to calculate the wilting rate, defined as the percentage of wilted plants relative to the total number of observed plants. A single seedling was defined as wilted when its leaves exhibited severe drooping, lost normal turgor pressure and became soft in texture; seedlings with only slightly curled leaf margins were not counted as wilted individuals.

For stomatal trait analysis, fully expanded leaves from the same nodal position on the main stem were excised. Epidermal peels were prepared using the transparent adhesive tape method: a small section of the abaxial epidermis was gently peeled off using tape, mounted on a glass slide, and observed under an optical microscope. Stomatal characteristics were imaged at magnifications ranging from 40× to 20×. Quantitative analysis was performed using ImageJ (v1.53t, National Institutes of Health, Bethesda, MD, USA) software. For each sample, ten random fields of view were selected to count the total number of stomata and the number of open stomata. SD was defined as the number of stomata per unit area (stomata/mm^2^), and the SOR was calculated as the ratio of open stomata to total stomata.

### 4.4. Determination Method of Structural Parameter Indices

Following drought treatment, fully expanded healthy leaves were harvested from each of the three selected replicate pots per treatment. Transverse sections were prepared from the middle region of each leaf blade, avoiding the central midrib to ensure consistent tissue composition across samples. Sections were observed and imaged under an optical microscope.

For each leaf sample, three well-prepared, intact transverse sections were randomly selected for quantification. For each section, three non-overlapping fields of view were randomly selected for measurement of LT, UEC, LEC, PT, ST, and VB using OPLENIC_Pro (v2.07; OPLENIC, Hangzhou, Zhejiang, China) software. This sampling scheme yielded 9 technical replicate measurements per leaf. For statistical analysis, the mean value of the 9 technical measurements was first calculated to generate a single anatomical trait value for each replicate pot. Pot-level values from the three biological replicates were then used for all subsequent variance and comparative analyses.

### 4.5. Determination Method of Water Physiological Index

Fresh whole plants by group were randomly excavated, wiped clean with alcohol, and weighed on an electronic balance to record their fresh weight (FW). The plants were then placed in envelopes and blanched at 105 °C for 30 min in an oven, followed by drying at 60 °C to constant weight. Dry weight (DW) was recorded, and WC was computed as WC = (FW − DW)/FW × 100%.

For the excised-leaf water loss rate assay, three fully expanded healthy leaves from the same nodal position were harvested from each of the three selected replicate pots (one leaf per plant, three plants per pot) as technical subsamples. The fresh weight (W_1_) of each leaf was immediately recorded after excision using a precision electronic balance with 0.1 mg readability. All leaves were placed individually in open Petri dishes and incubated in a controlled climate chamber set to a constant temperature of 25 ± 1 °C, relative humidity of 45 ± 5%, and dark conditions to eliminate interference from light and airflow fluctuations. Leaves were weighed sequentially at 20 min, 40 min, 1 h, 2 h, 4 h, and 8 h after excision to record the corresponding weight (W_2_). The water loss rate at each time point was calculated as RWL = (W_1_ − W_2_)/W_1_ × 100%. For statistical analysis, the mean RWL of the three subsampled leaves was first calculated to represent each replicate pot, and pot-level values from the three biological replicates were used for subsequent comparative analysis.

Leaves were randomly collected from the exact position of the main stem of plants by group while avoiding the central vein. Subsequently, 0.1 g of chopped leaf tissue was immersed in 10 mL of distilled water for 8 h, and the electrical conductivity R_1_ of the liquid was measured using a laboratory conductivity meter. The leaves were then subjected to a boiling water bath for 30 min. After allowing them to cool naturally to room temperature, the electrical conductivity R_2_ of the liquid was measured again, and the relative electrical conductivity was calculated as REC = R_1_/R_2_ × 100%.

### 4.6. Determination of Photosynthetic Physiological Index

The third to sixth mature and healthy leaves were randomly selected from the morphological upper part of plants by group. The LI-6800 portable photosynthesis measurement system (LI-COR Biosciences, Lincoln, NE, USA) was used, with the air flow rate set to 500 μmol·s^−1^, CO_2_ concentration to 500 μmol·mol^−1^, light intensity to 300 μmol·m^−2^·s^−1^, and temperature to 25 °C. Photosynthetic parameters were measured on-site between 9:00 and 10:00 a.m. After measurement, the leaves were collected to determine leaf area and calculate photosynthetic parameters (Pn, Tr, and Gs), and WUE = Pn/Tr.

### 4.7. Determination Method of Antioxidant Physiological Indices

In brief, 0.1 g of fresh leaves were randomly selected by group, rinsed with distilled water and dried, placed in 2.0 mL Eppendorf tubes, immediately plunged into liquid nitrogen for quick freezing for at least 4 h, and transferred to −80 °C freezer for storage pending testing. Total intracellular ROS was determined using the BBoxiProbe^®^ O08 fluorescent probe assay kit [64] (Bestbio, Shanghai, China). This kit can effectively reflect the overall oxidative stress level in plant tissues. Leaf samples preserved at −80 °C were thawed on ice. Briefly, 0.1 g leaf tissue was rinsed with pre-cooled pH 7.4 phosphate-buffered saline (PBS), homogenized in 1 mL ice-cold PBS, and centrifuged at 100× *g* for 5 min at 4 °C. A total of 190 µL supernatant was added into black 96-well plates, followed by mixing with 10 µL O08 working solution (stock solution diluted 10-fold with PBS). After fully blending, the mixture was incubated at 37 °C in darkness for 20–30 min. Subsequently, fluorescence intensity was instantly detected by a microplate reader at excitation wavelength of 516 nm and emission wavelength of 606 nm. Protein concentrations were normalized using the Bicinchoninic Acid (BCA) assay (Boxbio, Beijing, China, Cat. No. AKPR017). In this assay, proteins reduce Cu^2+^ to Cu^+^ under alkaline conditions; the generated Cu^+^ then chelates with BCA to form a purple complex with a characteristic absorbance peak at 562 nm. Finally, the total ROS level was normalized to the corresponding soluble protein content and expressed as RFU per mg protein. Additionally, MDA was assayed with a TBA colorimetric kit (Boxbio, Beijing, China, Cat. No. AKFA013M) following the manufacturer’s protocol. Absorbance was read at 450, 532 and 600 nm, MDA was quantified from the standard curve supplied with the kit, and results are expressed as nmol·g^−1^ FW [65]. The content of Pro was determined by the acidic ninhydrin method (Boxbio, Beijing, China, Cat. No. AKAM003M). After the samples were incubated in a water bath at 100 °C for 10 min, they were extracted with toluene, and the absorbance was measured at a wavelength of 520 nm [65]. The activity of POD was determined by the guaiacol method (Boxbio, Cat. Beijing, China, No. AKAO005M). The absorbance values at 470 nm at 30 s (A_1_) and 90 s (A_2_) were measured respectively, and the absorbance difference ΔA = A_2_ − A_1_ was calculated to determine the enzymatic reaction rate [66]. The activity of SOD was determined by the nitroblue tetrazolium (NBT) method (Boxbio, Beijing, China, Cat. No. AKAO001M-50S), and the enzyme activity was characterized by the inhibition rate of NBT photoreduction at a wavelength of 560 nm [67]. The activity of CAT was determined according to the UV spectrophotometry method. The absorbance values at 240 nm at 5 s (A_1_) and 65 s (A_2_) were measured respectively, and the absorbance difference ΔA = A_1_ − A_2_ was calculated. The enzyme activity was characterized by the decrease in absorbance of H_2_O_2_ [64] (Boxbio, Beijing, China, Cat. No. AKAO003-1M). Optical density (OD) values at specific wavelengths were measured using a microplate reader to determine ROS, MDA, and Pro contents, and POD, SOD, and CAT activities. Specific procedures were strictly followed in accordance with the kit instructions.

### 4.8. Comprehensive Evaluation of Drought Resistance

For all measured indicators including WR, PH, RL, SD, SOR, UEC, LEC, UC, LC, PT, ST, LT, VB, WC, REC, Pn, Tr, Gs, WUE, total ROS, MDA, Pro, POD, SOD, CAT and RWL, the drought resistance coefficient of each indicator was defined as the ratio of the measured value under drought stress to the measured value of the control before stress. The conversion of drought resistance coefficients was applied to eliminate differences in species background growth and basal indicator values, and to unify the baseline scale for drought resistance evaluation.

The original drought resistance coefficients include both positive and negative physiological indicators with inconsistent numerical change trends. Therefore, positive-oriented transformation was first performed on all drought resistance coefficients to unify the indicator direction, followed by Z-score standardization.

For positive indicators:

$$X_{ij}^{\prime }=(X_{ij}-X_{\min})/(X_{\max}-X_{\min})$$

For negative indicators:

$$X_{ij}^{\prime }=1-(X_{ij}-X_{\min})/(X_{\max}-X_{\min})$$

Z-score standardization was applied to the direction-unified drought resistance coefficient data. The standardization formula is as follows:

$$X_{ij}^{\prime \prime }=(X_{ij}^{\prime }-{\bar{X}}_{j})/S_{j}$$

where $X_{ij}^{\prime \prime }$ is the standardized value of the *j*-th indicator for the *i*-th species; $X_{ij}^{\prime }$ is the positively normalized drought resistance coefficient of the corresponding indicator; ${\bar{X}}_{j}$ is the sample mean of the *j*-th indicator; $S_{j}$ is the sample standard deviation of the *j*-th indicator.

PCA was performed on the standardized drought resistance coefficient dataset using SPSS software. Principal components with initial eigenvalues greater than 1 were extracted to achieve data dimensionality reduction and concentration of valid information. According to the component score coefficient matrix, the score of each species on each extracted principal component was calculated. The calculation formula is as follows:

$$F_{ik}=\sum_{j=1}^{n}\alpha_{kj}\cdot X_{ij}^{\prime \prime }$$

where $F_{ik}$ is the score of the *i*-th species on the *k*-th principal component; $\alpha_{kj}$ is the score coefficient of the j-th indicator in the *k*-th principal component; *n* is the total number of indicators involved in the evaluation.

The positive fuzzy membership function was used to normalize the scores of each principal component, mapping the principal component scores to the interval [0, 1]. The membership function value of each species corresponding to each principal component was calculated with the following formula:

$$U_{ik}=\frac{F_{ik}-F_{kmin}}{F_{kmax}-F_{kmin}}$$

where $U_{ik}$ is the membership function value of the i-th species on the *k*-th principal component; $F_{ik}$ is the score of the corresponding principal component for the species; $F_{k\max}$ and $F_{k\min}$ are the maximum and minimum scores of all samples on the *k*-th principal component, respectively.

The variance contribution rate of each principal component was used as the basis for weight assignment. The weight coefficient of each principal component was calculated with the following formula:

$$W_{k}=\frac{P_{k}}{\sum_{k=1}^{m}P_{k}}$$

where $W_{k}$ is the weight coefficient of the *k*-th principal component; $P_{k}$ is the variance contribution rate of the *k*-th principal component; m is the total number of extracted principal components; $\sum_{k=1}^{m}P_{k\;}$ is the cumulative variance contribution rate of all extracted principal components.

The weighted summation method was adopted to multiply the membership function value of each principal component by its corresponding weight and then sum the products, so as to obtain the comprehensive drought resistance evaluation value *D* of each species. The calculation formula is as follows:

$$D_{i}=\sum_{k=1}^{m}(U_{ik}\cdot W_{k})$$

where $D_{i}$ is the comprehensive drought resistance evaluation value of the *i*-th species.

### 4.9. Data Processing and Analysis

All experimental data were compiled and standardized using Microsoft Excel 2021 to ensure comparability across all samples. Prior to parametric statistical analysis, the Shapiro–Wilk test and Levene’s test were performed to verify the normality of data distribution and homogeneity of variances, respectively. All datasets satisfied the assumptions of parametric tests (*p* > 0.05).

Full-factorial two-way ANOVA was first performed for all 26 traits to test the main effects of species, drought duration and their interaction (complete results in Supplementary Table S8). Given the widespread significant Species × Drought duration interactions, simple effect tests via stratified one-way ANOVA with Tukey’s HSD post hoc comparison were further conducted using SPSS 26.0 at a significance level of *p* < 0.05, to resolve interspecific differences at each drought stage and temporal variation within each species. Different uppercase letters in figures indicate significant interspecific differences under the same treatment, while different lowercase letters indicate significant temporal differences within the same species.

All experimental figures were plotted with Origin 2018 software.

## 5. Conclusions

Multivariate trait response analysis revealed that the three studied *Zygophyllum* species employ a suite of key strategies to adapt to arid environments, including stomatal regulation to minimize water loss, osmolyte accumulation to stabilize cellular structures, and enhanced antioxidant activity to mitigate oxidative damage. Quantitative evaluation using the membership function model indicated that the drought resistance ranking at the seedling stage is *Z. brachypterum* (0.649) > *Z. fabago* (0.592) > *Z. obliquum* (0.535). This study provides comparative data regarding the physiological ecology of *Zygophyllum* species and offers a quantitative evaluation of drought resistance among these three closely related taxa. In contrast to previous studies that have focused on single species or morphological descriptions alone, the present work integrates multiple physiological indicators to help reveal interspecific differences in adaptive coordination. These findings support further research on the stress resistance mechanisms of xerophytes and help improve comprehensive evaluation systems for plant drought resistance.

## Figures and Tables

**Figure 1:**
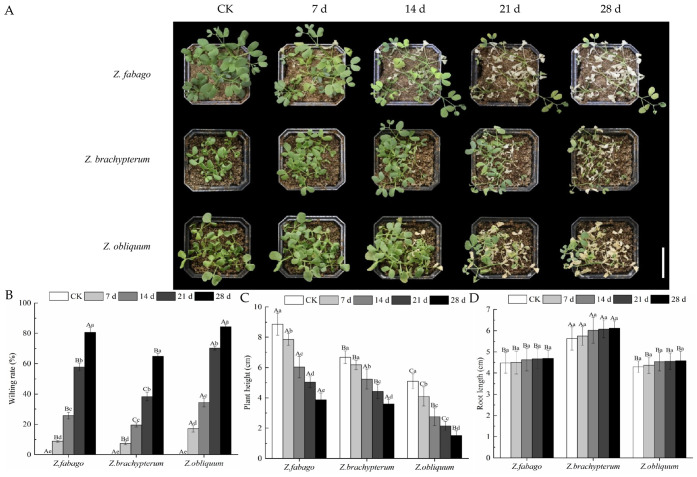
Wilting rate, plant height, and root length of three species of *Zygophyllum* under drought stress. (**A**) Wilting phenotypes of three *Zygophyllum* species under drought stress. (Scale bar = 5 cm) (**B**) Statistical analysis of the wilting rate. (**C**) Statistical analysis of plant height. (**D**) Statistical analysis of root length. Values are means ± standard deviation. Different uppercase letters indicate significant differences among species under the same stress treatment, and different lowercase letters indicate significant differences among stress treatments within the same species (*p* < 0.05).

**Figure 2:**
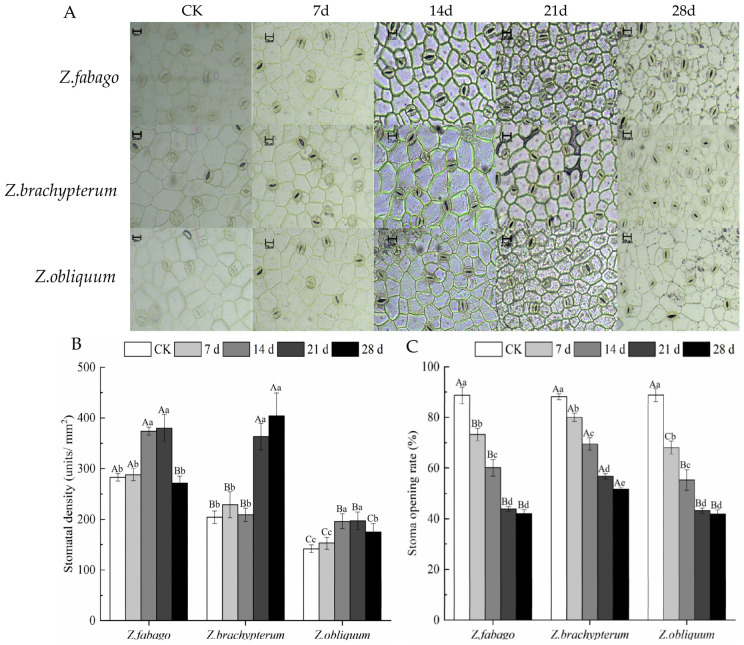
SD and SOR of three *Zygophyllum* species under drought stress. (**A**) Effects of 20% PEG-6000 treatment on stomatal density and aperture ratio in three *Zygophyllum* species. (**B**) Statistical analysis of SD. (**C**) Statistical analysis of SOR. Values are means ± standard deviation. Different uppercase letters indicate significant differences among species under the same stress treatment, and different lowercase letters indicate significant differences among stress treatments within the same species (*p* < 0.05).

**Figure 3:**
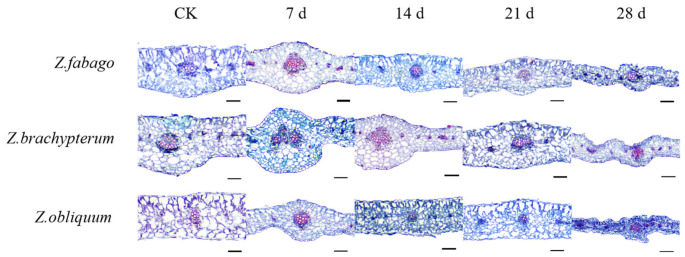
Leaf anatomical structure of three *Zygophyllum* species under drought stress. Sections were stained with safranin-fast green: red indicates lignified xylem tissue, and blue-green indicates parenchyma and mesophyll cells. All scales in the figure are 100 μm.

**Figure 4:**
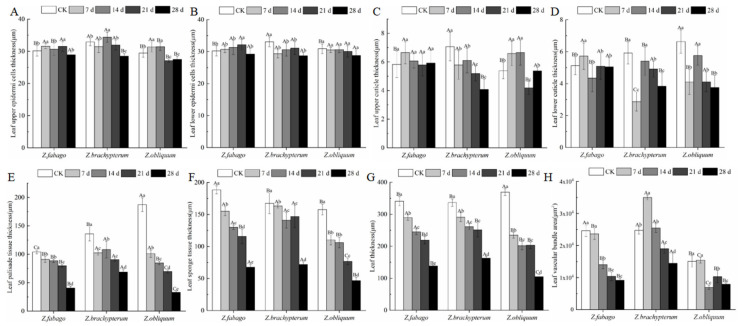
Leaf anatomical structure of three *Zygophyllum* species under drought stress. (**A**) Statistical analysis of UEC. (**B**) Statistical analysis of LEC. (**C**) Statistical analysis of UC. (**D**) Statistical analysis of LC. (**E**) Statistical analysis of PT. (**F**) Statistical analysis of ST. (**G**) Statistical analysis of LT. (**H**) Statistical analysis of VB. Values are means ± standard deviation. Different uppercase letters indicate significant differences among species under the same stress treatment, and different lowercase letters indicate significant differences among stress treatments within the same species (*p* < 0.05).

**Figure 5:**
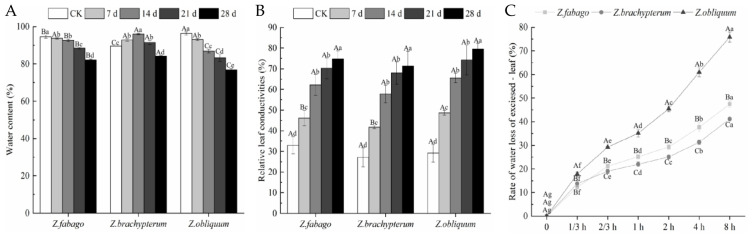
Water physiological characteristics of three Zygophyllum species under drought stress. (**A**) Analysis of water content. (**B**) Analysis of relative conductivity. (**C**) Analysis of water loss rate. Values are means ± standard deviation. Different uppercase letters indicate significant differences among species under the same stress treatment, and different lowercase letters indicate significant differences among stress treatments within the same species (*p* < 0.05).

**Figure 6:**
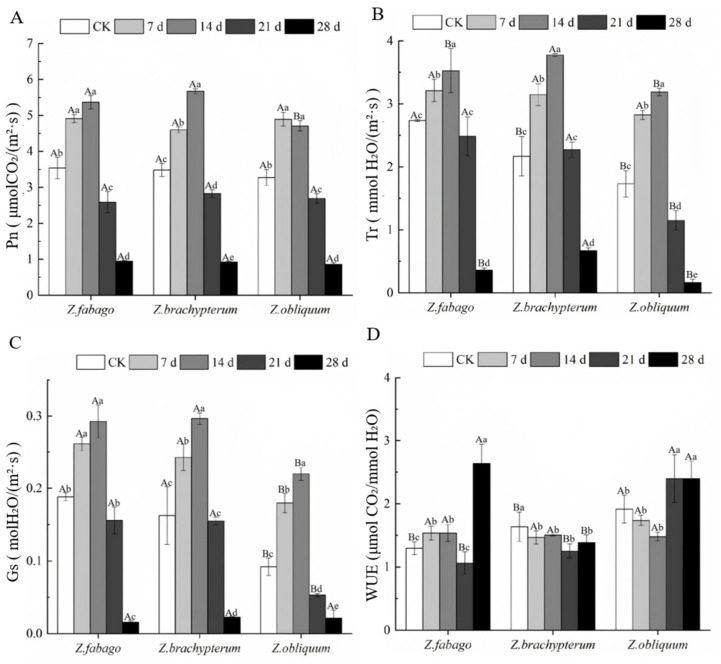
Photosynthetic physiological characteristics of three *Zygophyllum* species under drought stress. (**A**) Analysis of Pn. (**B**) Analysis of Tr. (**C**) Analysis of Gs. (**D**) Analysis of WUE. Values are means ± standard deviation. Different uppercase letters indicate significant differences among species under the same stress treatment, and different lowercase letters indicate significant differences among stress treatments within the same species (*p* < 0.05).

**Figure 7:**
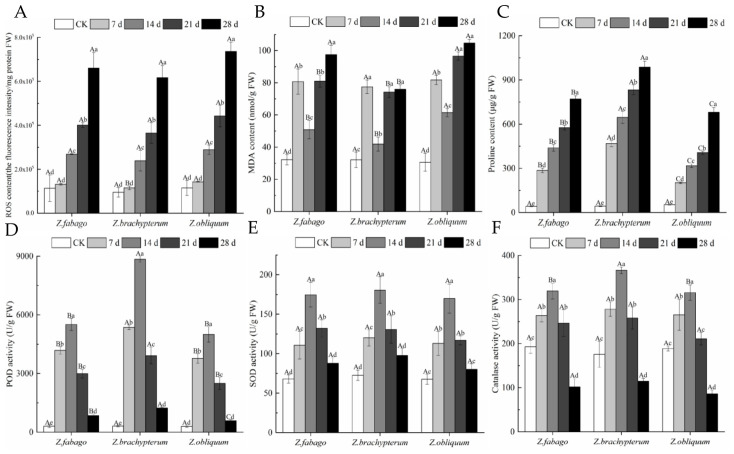
Antioxidant physiological characteristics of three *Zygophyllum* species under drought stress. (**A**) Analysis of total ROS content. (**B**) Analysis of MDA content. (**C**) Analysis of Pro content. (**D**) Analysis of POD activity. (**E**) Analysis of SOD activity. (**F**) Analysis of CAT activity. Values are means ± standard deviation. Different uppercase letters indicate significant differences among species under the same stress treatment, and different lowercase letters indicate significant differences among stress treatments within the same species (*p* < 0.05).

**Figure 8:**
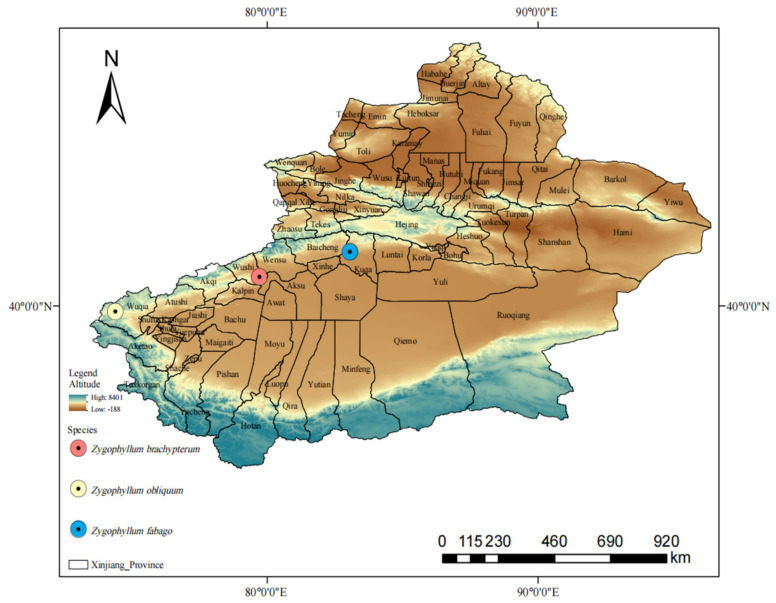
Location of sampling site (Xinjiang Uygur Autonomous Region, China).

**Table 1:** Principal component loading matrix, eigenvalues, variance contribution rates and cumulative variance contribution rates of the first five principal components.

Index	Variable	Principal Components				
		PC1	PC2	PC3	PC4	PC5
WR	x1	0.070	−0.032	−0.002	0.088	−0.006
PH	x2	0.057	−0.029	−0.126	−0.021	−0.412
RL	x3	0.005	0.070	−0.308	0.037	0.437
SD	x4	0.009	−0.077	0.267	0.295	0.369
SOR	x5	0.063	−0.084	−0.048	0.135	0.049
UEC	x6	0.055	0.091	−0.022	−0.102	0.131
LEC	x7	0.044	0.002	0.071	−0.380	0.024
UC	x8	0.043	0.017	0.182	−0.064	−0.241
LC	x9	0.032	−0.065	0.127	−0.388	0.225
PT	x10	0.057	−0.078	0.011	−0.038	0.362
ST	x11	0.068	−0.026	−0.086	−0.001	−0.082
LT	x12	0.066	−0.070	−0.039	0.001	0.053
VB	x13	0.050	0.004	−0.195	0.288	−0.030
WC	x14	0.066	0.018	−0.032	0.026	0.043
REC	x15	0.061	−0.105	−0.015	0.099	−0.009
Pn	x16	0.058	0.093	0.131	0.122	−0.024
Tr	x17	0.060	0.110	0.056	0.022	−0.118
Gs	x18	0.057	0.117	0.047	0.048	−0.160
WUE	x19	−0.043	−0.079	0.178	0.186	0.137
ROS	x20	0.069	−0.018	0.047	0.087	−0.042
MDA	x21	0.057	−0.049	−0.034	−0.149	0.244
Pro	x22	−0.048	0.103	−0.196	−0.042	0.153
POD	x23	0.025	0.187	0.047	0.123	0.154
SOD	x24	0.011	0.197	0.081	−0.059	0.130
CAT	x25	0.046	0.149	0.094	0.021	0.081
RWL	x26	0.063	−0.074	−0.105	−0.071	0.028
Eigenvalue	/	13.888	4.739	2.427	1.595	1.040
Contribution rate (%)	/	53.414	18.227	9.333	6.135	4.000
Cumulative contribution rate (%)	/	53.414	71.641	80.974	87.109	91.109
Weight	/	0.586	0.200	0.102	0.067	0.044

**Table 2:** Membership function values and comprehensive evaluation values of each indicator.

Species	U(PC1)	U(PC2)	U(PC3)	U(PC4)	U(PC5)	*D*
*Z. fabago*	0.670	0.566	0.562	0.331	0.140	0.592
*Z. brachypterum*	0.731	0.637	0.315	0.485	0.653	0.649
*Z. obliquum*	0.511	0.464	0.815	0.508	0.578	0.535

## Data Availability

The original contributions presented in the study are included in this article, and further inquiries can be directed to the corresponding authors.

## Supplementary Materials

The following supporting information can be downloaded at: https://www.mdpi.com/article/10.3390/plants15152317/s1, Table S1. Apparent morphological characteristics of three species of *Zygophyllum* under drought stress; Table S2. Leaf anatomical structure characteristics of three species of *Zygophyllum* under drought stress; Table S3. Characteristics of water content and relative electrical conductivity of three species of *Zygophyllum* under drought stress; Table S4. Characteristics of water loss rate of leaves in vitro of three species of *Zygophyllum*; Table S5. Photosynthetic physiological characteristics of three species of *Zygophyllum* under drought stress; Table S6. Antioxidant physiological characteristics of three *Zygophyllum* plants under drought stress; Table S7. Basic information of each sampling site; Table S8. Two-way ANOVA results for all 26 measured functional traits in three *Zygophyllum* species, testing the main effects of species, drought duration, and their interaction.

## Abbreviations

The following abbreviations are used in this manuscript:

Pro	Proline
Gly	Glycine
ROS	Reactive oxygen species
MDA	Malondialdehyde
POD	Peroxidase
SOD	Superoxide dismutase
CAT	Catalase
FW	Fresh weight
DW	Dry weight
WC	Water content
RWL	Rate of excised-leaf water loss
REC	Relative electrical conductivity
Pn	Photosynthetic rate
Tr	Transpiration rate
Gs	Stomatal conductivity
WUE	Water use efficiency
PCA	Principal components analysis
PH	Plant height
RL	Root length
WR	Wilting rate
SD	Stomatal density
SOR	Stoma opening rate
LT	Leaf thickness
ST	Leaf sponge tissue thickness
PT	Leaf palisade tissue thickness
VB	Leaf vascular bundle area
UEC	Leaf upper epidermis cell thickness
LEC	Leaf lower epidermis cell thickness
UC	Leaf upper cuticle thickness
LC	Leaf lower cuticle thickness
