# Supplementary material for: Morphological and Physiological Responses to Drought Stress of Three Species of the Zygophyllum Genus and a Comprehensive Evaluation of Their Drought Resistance
Source: Plants (Basel). 2026 Jul 28;15(15):2317. doi: 10.3390/plants15152317 (PMC13467695; doi:10.3390/plants15152317)
Supplement: Supplementary file 1 [file plants-15-02317-s001.zip › plants-4376070-supplementary.pdf]

Supplemental information：

Table S1 Apparent morphological characteristics of three species of *Zygophyllum* under drought stress

Index	Species	Stress time				
		CK	7 d	14 d	21 d	28 d
Wilting rate (%)	*Z. fabago*	0±0 Ae	8.907±0.592 Bd	25.764±2.303 Bc	57.844±2.494 Bb	80.717±2.968 Aa
	Z. brachypterum	0±0 Ae	7.472±1.049 Bd	19.562±1.427 Cc	38.326±2.913 Cb	64.879±1.874 Ba
	Z. obliquum	0±0 Ae	17.058±2.087 Ad	34.348±2.883 Ac	70.274±1.277 Ab	84.324±1.506 Aa
Plant height (cm)	*Z. fabago*	8.556±0.73 Aa	7.856±0.392 Ab	6.033±0.707 Ac	5.033±0.365 Ad	3.867±0.45 Ae
	Z. brachypterum	6.678±0.426 Ba	6.178±0.312 Ba	5.233±0.662 Ab	4.422±0.483 Bc	3.6±0.294 Ad
	Z. obliquum	5.089±0.5 Ca	4.1±0.631 Cb	2.756±0.581 Bc	2.156±0.299 Cc	1.511±0.314 Bd
Root length (cm)	*Z. fabago*	4.478±0.485 Ba	4.5±0.542 Ba	4.633±0.537 Ba	4.678±0.476 Ba	4.7±0.383 Ba
	Z. brachypterum	5.633±0.546 Aa	5.744±0.437 Aa	6.022±0.612 Aa	6.078±0.418 Aa	6.111±0.354 Aa
	Z. obliquum	4.3±0.279 Ba	4.367±0.386 Ba	4.533±0.435 Ba	4.567±0.383 Ba	4.589±0.451 Ba
Stomatal density (units/mm^2)	*Z. fabago*	283±7.483 Ab	288±12.083 Ab	374±8 Aa	380±27.019 Aa	271±13.928 Bb
	Z. brachypterum	204±12.41 Bb	229±25.768 Bb	209±13.191 Bb	363±26.382 Aa	404±45.211 Aa
	Z. obliquum	142±7.483 Cc	153±11.662 Cc	196±14.629 Ba	197±17.205 Ba	175±16.733 Cb
Stoma opening rate (%)	*Z. fabago*	88.734±3.263 Aa	73.204±2.479 Bb	60.115±3.274 Bc	43.899±0.971 Bd	42.036±1.583 Bd
	Z. brachypterum	88.199±1.174 Aa	80.018±1.687 Ab	69.508±2.415 Ac	56.766±1.11 Ad	51.699±0.739 Ae
	Z. obliquum	88.763±2.502 Aa	68.071±2.524 Cb	55.301±4.028 Bc	43.117±1.099 Bd	41.834±1.608 Bd

Note: Values are means ± standard deviation. Different uppercase letters indicate significant differences among species under the same stress treatment, and different lowercase letters indicate significant differences among stress treatments with in the same species ($P < 0.05$).

Table S2 Leaf anatomical structure characteristics of three species of *Zygophyllum* under drought stress

Index	Species	Stress time				
		CK	7 d	14 d	21 d	28 d
UEC (μm)	*Z. fabago*	30.155±1.623 Bb	31.568±0.725 Aa	30.623±1.919 Bb	31.523±1.513 Aa	28.912±0.886 Ab
	Z. brachypterum	32.885±1.142 Ab	31.475±1.889 Ab	34.383±1.556 Aa	31.967±1.541 Ab	28.473±0.832 Bc
	Z. obliquum	29.472±1.357 Bb	31.348±1.569 Aa	31.453±1.233 Ba	27.08±0.494 Bc	27.452±0.977 Bc
LEC (μm)	*Z. fabago*	30.223±1.548 Bb	30.69±1.016 Ab	31.317±2.226 Ab	32.16±1.585 Aa	29.223±1.762 Ab
	Z. brachypterum	33.052±1.535 Aa	29.317±1.301 Ab	30.623±1.983 Ab	31.125±1.996 Ab	28.705±0.976 Ab
	Z. obliquum	30.898±1.515 Ba	30.503±0.813 Aa	30.628±0.972 Aa	30.088±1.774 Aa	28.767±1.844 Aa
UC (μm)	*Z. fabago*	5.827±0.932 Ba	6.65±0.771 Aa	6.068±0.485 Aa	5.792±0.774 Aa	5.92±0.874 Aa
	Z. brachypterum	7.07±0.997 Aa	5.793±0.993 Ab	6.108±0.87 Ab	5.19±0.526 Ac	4.082±0.73 Bc
	Z. obliquum	5.392±0.57 Bb	6.592±0.847 Aa	6.648±0.866 Aa	4.178±0.445 Bc	5.375±0.244 Ab
LC (μm)	*Z. fabago*	5.12±0.557 Bb	5.722±0.831 Aa	4.352±0.868 Bb	5.087±0.812 Ab	5.055±0.57 Ab
	Z. brachypterum	5.92±0.684 Ba	2.873±0.601 Cc	5.407±0.878 Ba	4.915±0.498 Ab	3.837±0.845 Bc
	Z. obliquum	6.633±0.724 Aa	4.107±0.782 Bb	5.757±0.601 Aa	4.11±0.616 Ab	3.757±0.747 Bb
PT (μm)	*Z. fabago*	104.082±3.301 Ca	90.925±5.225 Bb	88.413±3.081 Bb	79.713±2.342 Bc	40.55±3.139 Bd
	Z. brachypterum	135.938±12.638 Ba	102.595±4.301 Ab	108.33±14.556 Ac	90.528±2.641 Ac	68.87±4.148 Ad
	Z. obliquum	187.262±12.305 Aa	101.157±6.629 Ab	84.883±3.029 Bc	69.73±2.71 Cd	32.983±2.535 Ce
ST (μm)	*Z. fabago*	188.393±6.454 Aa	155.097±7.086 Ab	129.95±3.293 Ac	115.648±11.325 Bd	67.548±4.23 Ae
	Z. brachypterum	167.315±16.137 Ba	163.183±3.129 Ab	140.788±12.051 Ac	146.768±17.39 Ac	71.772±3.737 Ad
	Z. obliquum	157.617±8.547 Ba	110.078±7.74 Bb	106.018±8.316 Bb	76.618±5.264 Cc	46.44±5.322 Bd
LT (μm)	*Z. fabago*	340.737±14.349 Ba	289.43±8.188 Ab	244.85±8.926 Ac	219.038±11.939 Bd	138.39±2.837 Be
	Z. brachypterum	336.607±11.548 Ba	290.72±13.708 Ab	260.955±8.799 Ac	251.098±22.409 Bc	162.693±6.453 Ad
	Z. obliquum	369.337±10.515 Aa	234.722±9.283 Bb	201.79±12.955 Bc	203.06±10.114 Bc	104.418±8.146 Cd
VB (μm^2)	*Z. fabago*	24638.158±1806.54 Aa	23637.505±1742.31 Ba	14055.278±1314.03 Bb	10400.25±1346.59 Bc	9168.947±665.38 Bc
	Z. brachypterum	24711.553±1099.31 Ab	34983.765±712.995 Aa	25459.09±1359.08 Ab	18989.203±1465.9 Ac	14472.125±3009.56 Ad
	Z. obliquum	15075.97±1776.2 Ba	15453.583±877.86 Ca	6984.752±671.59 Cc	10301.118±1884.11 Bb	7903.718±494.91 Bc

Note: Values are means ± standard deviation. Different uppercase letters indicate significant differences among species under the same stress treatment, and different lowercase letters indicate significant differences among stress treatments with in the same species ($P < 0.05$).

Table S3 Characteristics of water content and relative electrical conductivity of three species of *Zygophyllum* under drought stress

Index	Species	Stress time				
		CK	7 d	14 d	21 d	28 d
WC (%)	*Z. fabago*	94.589±0.887 Ba	93.71±0.415 Ab	92.685±0.635 Bb	88.431±0.426 Bc	82.088±0.568 Bd
	Z. brachypterum	89.526±0.034 Cc	92.846±0.646 Ab	96.054±0.297 Aa	91.584±1.048 Ab	84.164±0.217 Ad
	Z. obliquum	96.387±0.869 Aa	93.082±0.527 Ab	86.889±1.005 Cc	83.217±1.8 Cd	76.815±0.724 Ce
REC (%)	*Z. fabago*	32.993±4.135 Ac	46.137±3.784 Bb	62.183±4.998 Aa	70.236±5.063 Aa	74.783±3.892 Aa
	Z. brachypterum	27.131±4.581 Ad	41.725±0.715 Bc	57.84±4.261 Ab	67.923±5.501 Ab	71.317±7.108 Aa
	Z. obliquum	29.163±4.261 Ad	48.714±1.124 Ac	65.566±2.329 Ab	74.288±7.247 Ab	79.554±2.711 Aa

Note: Values are means ± standard deviation. Different uppercase letters indicate significant differences among species under the same stress treatment, and different lowercase letters indicate significant differences among stress treatments with in the same species ($P < 0.05$).

Table S4 Characteristics of water loss rate of leaves in vitro of three species of *Zygophyllum* under drought stress

Species	Drying time						
	0	1/3 h	2/3 h	1 h	2 h	4 h	8 h
Z. fabago	0±0 Ag	12.23±0.71 Bf	21.19±0.91 Be	25.14±1 Bd	29.29±1.1 Bc	37.67±1.94 Bb	47.35±1.38 Ba
Z. brachypterum	0±0 Ag	13.77±1.32 Bf	19.02±0.92 Ce	21.99±1 Cd	25.08±0.8 Cc	31.3±0.95 Cb	41.16±0.43 Ca
Z. obliquum	0±0 Ag	17.86±0.76 Af	29.22±0.31 Ae	35.16±1.57 Ad	45.47±1.24 Ac	60.96±1.94 Ab	75.84±2.13 Aa

Note: Values are means ± standard deviation. Different uppercase letters indicate significant differences among species under the same stress treatment, and different lowercase letters indicate significant differences among stress treatments with in the same species ($P < 0.05$).

Table S5 Photosynthetic physiological characteristics of three species of *Zygophyllum* under drought stress

Index	Species	Stress time				
		CK	7 d	14 d	21 d	28 d
Pn (μmol CO_2/(m^2·s))	*Z. fabago*	3.54±0.297 Ab	4.911±0.11 Aa	5.366±0.19 Aa	2.585±0.299 Ac	0.941±0.027 Ad
	Z. brachypterum	3.48±0.183 Ac	4.599±0.085 Ab	5.67±0.067 Aa	2.825±0.106 Ad	0.922±0.019 Ae
	Z. obliquum	3.272±0.219 Ab	4.894±0.189 Aa	4.707±0.142 Ba	2.693±0.133 Ac	0.862±0.046 Ad
Tr (mmol H_2O/(m^2·s))	*Z. fabago*	2.734±0.016 Ac	3.21±0.178 Ab	3.525±0.349 Ba	2.483±0.308 Ac	0.361±0.032 Bd
	Z. brachypterum	2.168±0.314 Bc	3.145±0.173 Ab	3.776±0.023 Aa	2.27±0.123 Ac	0.669±0.042 Ad
	Z. obliquum	1.731±0.21 Bc	2.823±0.072 Ab	3.185±0.059 Ba	1.149±0.155 Bd	0.163±0.054 Be
Gs (mol H_2O/(m^2·s))	*Z. fabago*	0.188±0.005 Ab	0.261±0.01 Aa	0.293±0.023 Aa	0.156±0.018 Ab	0.016±0.002 Ac
	Z. brachypterum	0.162±0.04 Ac	0.242±0.018 Ab	0.297±0.008 Aa	0.155±0.005 Ac	0.023±0.001 Ad
	Z. obliquum	0.092±0.012 Bc	0.18±0.013 Bb	0.22±0.009 Ba	0.053±0.002 Bd	0.021±0.011 Ae
Wue (μmol CO_2/mmol H_2O)	*Z. fabago*	1.294±0.102 Bc	1.536±0.109 Ab	1.535±0.136 Ab	1.059±0.178 Bc	2.634±0.306 Aa
	Z. brachypterum	1.635±0.23 Ba	1.468±0.103 Ab	1.502±0.014 Ab	1.251±0.114 Bb	1.385±0.119 Bb
	Z. obliquum	1.913±0.219 Ab	1.735±0.08 Ab	1.479±0.068 Ab	2.393±0.377 Aa	2.394±0.277 Aa

Note: Values are means ± standard deviation. Different uppercase letters indicate significant differences among species under the same stress treatment, and different lowercase letters indicate significant differences among stress treatments with in the same species ($P < 0.05$).

Table S6 Antioxidant physiological characteristics of three *Zygophyllum* plants under drought stress

Index	Species	Stress time				
		CK	7 d	14 d	21 d	28 d
ROS (the fluorescence intensity/mg protein)	*Z. fabago*	113575.5±60209.8 Ad	131870.9±4083.6 Ad	268597±2388.8 Ac	400553.5±10787.5 Ab	659981.5±71112.5 Aa
	Z. brachypterum	94835±21787.4 Ad	115230.9±8631.6 Bd	238683±46934 Ac	364763±46888.1 Ab	616450.7±55278 Aa
	Z. obliquum	114727.6±34049.3 Ad	142881.9±1692.6 Ad	288923±20958.4 Ac	442172.7±49062.2 Ab	735563.3±42627.9 Aa
MDA (nmol/g)	*Z. fabago*	32.2±3.2 Ad	80.736±7.8 Ab	50.9±5.7 Bc	81.1±3.7 Bb	97.5±5.8 Aa
	Z. brachypterum	32.1±4.7 Ac	77.5±4.2 Aa	42±4.3 Bb	74.2±3.3 Ba	75.9±2.7 Ba
	Z. obliquum	30.6±5.6 Ad	81.8±2.9 Ab	61.4±2.6 Ac	96.6±2.5 Aa	104.7±2.3 Aa
Pro (μg/g)	*Z. fabago*	42±1.9 Ae	286.3±15.3 Bd	439.1±22.5 Bc	575.6±16.5 Bb	770.7±21.6 Ba
	Z. brachypterum	43.1±2.3 Ae	467.7±20.3 Ad	645.8±40.3 Ac	832.9±33.5 Ab	987.7±39.9 Aa
	Z. obliquum	53.2±1.8 Ae	202±5.8 Cd	317.2±12.7 Cc	406.7±10.4 Cb	680.3±32.2 Ca
POD (U/g)	*Z. fabago*	304.9±63.7 Ae	4183.9±189.8 Bb	5511.8±308.8 Ba	2991.1±228.5 Bc	847.5±81.3 Bd
	Z. brachypterum	307.2±31.6 Ae	5357.2±116.7 Ab	8848.2±154.6 Aa	3914.7±425.6 Ac	1235.7±57.4 Ad
	Z. obliquum	295.5±47.3 Ad	3776.4±243.7 Bb	4996.6±391.2 Ba	2499.3±312.6 Bc	590.7±48 Cd
SOD (U/g)	*Z. fabago*	67.9±5.1 Ad	110.7±17.5 Ac	174.6±15.6 Aa	132±10.9 Ab	87.7±6.9 Ad
	Z. brachypterum	72.6±6.5 Ad	120±10.1 Ac	180.6±16.7 Aa	130.7±17.4 Ab	97.8±9 Ad
	Z. obliquum	67.6±6.3 Ac	113.1±15.1 Ab	169.8±18.2 Aa	116.9±5.8 Ab	80.3±6.8 Ac
CAT (U/g)	*Z. fabago*	192.8±14.9 Ac	263.7±14.2 Ab	319.2±17.7 Ba	246.6±30.156 Ab	101.7±18.6 Ad
	Z. brachypterum	175.7±29.3 Ac	278±16.3 Ab	366.2±7.2 Aa	258.1±25 Ab	114.8±6.3 Ad
	Z. obliquum	188.7±5.7 Ac	265.3±35.4 Ab	315.6±17.6 Ba	211±14.3 Ac	85.8±8.3 Ad

Note: Values are means ± standard deviation. Different uppercase letters indicate significant differences among species under the same stress treatment, and different lowercase letters indicate significant differences among stress treatments with in the same species ($P < 0.05$).

Table S7 Basic information of each sampling site

Species	Latitude/°N	Longitude/°E	Elevation/m
Z. fabago	41°53′32.27″	83°3′30.58″	1295
Z. brachypterum	41°6′49.81″	79°49′14.69″	1209
Z. obliquum	39°49′51.34″	74°33′25.58″	2746

Table S8 Two-way ANOVA results for all 26 measured functional traits in three *Zygophyllum* species, testing the main effects of species, drought duration, and their interaction

Index	Source of variation	df	Mean Square	*F*-value	*P*-value	Significance
WR	Species	2	892.685	502.445	<0.001	***
	Drought duration	4	9165.294	5158.659	<0.001	***
	Species × Drought duration	8	118.789	66.860	<0.001	***
PH	Species	2	39.683	179.981	<0.001	***
	Drought duration	4	23.545	106.788	<0.001	***
	Species × Drought duration	8	0.535	2.424	0.038	*
RL	Species	2	6.436	28.632	<0.001	***
	Drought duration	4	0.688	3.060	0.032	*
	Species × Drought duration	8	0.042	0.187	0.991	ns
SD	Species	2	76790.220	465.865	<0.001	***
	Drought duration	4	13150.122	79.778	<0.001	***
	Species × Drought duration	8	8697.640	52.766	<0.001	***
SOR	Species	2	425.261	86.904	<0.001	***
	Drought duration	4	3039.350	621.104	<0.001	***
	Species × Drought duration	8	38.438	7.855	<0.001	***
UEC	Species	2	19.650	6.936	0.003	**
	Drought duration	4	13.330	4.705	0.005	**
	Species × Drought duration	8	13.038	4.602	0.001	**
LEC	Species	2	1.703	0.675	0.517	ns
	Drought duration	4	7.285	2.887	0.039	*
	Species × Drought duration	8	5.918	2.345	0.043	*

	Species	2	1.257	2.082	0.142	ns
UC	Drought duration	4	5.075	8.408	<0.001	***
	Species × Drought duration	8	1.666	2.760	0.021	*
	Species	2	2.193	4.742	0.016	*
LC	Drought duration	4	4.964	10.732	<0.001	***
	Species × Drought duration	8	3.509	7.585	<0.001	***
	Species	2	2036.658	76.733	<0.001	***
PT	Drought duration	4	10648.948	401.207	<0.001	***
	Species × Drought duration	8	1549.303	58.371	<0.001	***
	Species	2	6729.606	59.556	<0.001	***
ST	Drought duration	4	14592.715	129.144	<0.001	***
	Species × Drought duration	8	721.402	6.384	<0.001	***
	Species	2	5348.181	29.140	<0.001	***
LT	Drought duration	4	58520.994	318.852	<0.001	***
	Species × Drought duration	8	1474.531	8.034	<0.001	***
	Species	2	530820267.200	425.191	<0.001	***
VB	Drought duration	4	314910692.800	252.246	<0.001	***
	Species × Drought duration	8	38829801.900	31.103	<0.001	***
	Species	2	56.605	121.282	<0.001	***
WC	Drought duration	4	244.364	523.569	<0.001	***
	Species × Drought duration	8	37.231	79.770	<0.001	***
	Species	2	355.887	27.387	<0.001	***
REC	Drought duration	4	3304.548	254.303	<0.001	***
	Species × Drought duration	8	6.971	0.536	0.820	ns
WLR	Species	2	1555.051	1394.983	<0.001	***
	Drought duration	4	3784.630	3395.062	<0.001	***

	Species × Drought duration	8	156.974	140.816	<0.001	***
	Species	2	0.327	14.350	<0.001	***
Pn	Drought duration	4	27.903	1224.588	<0.001	***
	Species × Drought duration	8	0.188	8.269	<0.001	***
	Species	2	1.383	33.287	<0.001	***
Tr	Drought duration	4	12.985	312.534	<0.001	***
	Species × Drought duration	8	0.236	5.691	<0.001	***
	Species	2	0.020	167.715	<0.001	***
Gs	Drought duration	4	0.088	729.247	<0.001	***
	Species × Drought duration	8	0.003	25.886	<0.001	***
	Species	2	0.697	10.450	<0.001	***
Wue	Drought duration	4	0.424	6.360	0.001	**
	Species × Drought duration	8	0.455	6.816	<0.001	***
	Species	2	13730255050	12.472	<0.001	***
ROS	Drought duration	4	497338318400	451.750	<0.001	***
	Species × Drought duration	8	6668607909	6.057	<0.001	***
	Species	2	891.096	43.609	<0.001	***
MDA	Drought duration	4	5980.432	292.671	<0.001	***
	Species × Drought duration	8	147.110	7.199	<0.001	***
	Species	2	261803.730	340.119	<0.001	***
Pro	Drought duration	4	737005.498	957.473	<0.001	***
	Species × Drought duration	8	21361.159	27.751	<0.001	***
	Species	2	9665622.331	354.987	<0.001	***
POD	Drought duration	4	55902357.660	2053.113	<0.001	***
	Species × Drought duration	8	2137388.445	78.499	<0.001	***
SOD	Species	2	1294.792	8.080	0.002	**

	Drought duration	4	16664.942	103.997	<0.001	***
	Species × Drought duration	8	143.188	0.894	0.534	ns
	Species	2	3843.749	11.546	<0.001	***
CAT	Drought duration	4	70170.006	210.776	<0.001	***
	Species × Drought duration	8	1380.180	4.146	0.003	**

Note: Significance codes: ns, not significant ($P \geq 0.05$); *, $P < 0.05$; **, $P < 0.01$; ***, $P < 0.001$.
